# High Colonization Rate and Heterogeneity of ESBL- and Carbapenemase-Producing *Enterobacteriaceae* Isolated from Gull Feces in Lisbon, Portugal

**DOI:** 10.3390/microorganisms8101487

**Published:** 2020-09-28

**Authors:** Marta Aires-de-Sousa, Claudine Fournier, Elizeth Lopes, Hermínia de Lencastre, Patrice Nordmann, Laurent Poirel

**Affiliations:** 1 Emerging Antibiotic Resistance Unit, Medical and Molecular Microbiology, Faculty of Science and Medicine, University of Fribourg, CH-1700 Fribourg, Switzerland; msousa@esscvp.eu (M.A.-d.-S.); claudine.fournier@unifr.ch (C.F.); patrice.nordmann@unifr.ch (P.N.); 2 Escola Superior de Saúde da Cruz Vermelha Portuguesa, 1300 Lisbon, Portugal; 3 Laboratory of Molecular Genetics, Instituto de Tecnologia Química e Biológica António Xavier, Universidade Nova de Lisboa, 2780 Oeiras, Portugal; elizeth.lopes08@gmail.com (E.L.); lencash@mail.rockefeller.edu (H.d.L.); 4 Laboratory of Microbiology and Infectious Diseases, The Rockefeller University, New York, NY 10065, USA; 5 Swiss National Reference Center for Emerging Antibiotic Resistance (NARA), CH-1700 Fribourg, Switzerland

**Keywords:** carbapenemase, gulls, Portugal, ESBL, *Enterobacteriaceae*

## Abstract

In order to evaluate whether seagulls living on the Lisbon coastline, Portugal, might be colonized and consequently represent potential spreaders of multidrug-resistant bacteria, a total of 88 gull fecal samples were screened for detection of extended-spectrum β-lactamase (ESBL)- or carbapenemase-producing *Enterobacteriaceae* for methicillin-resistant *Staphylococcus aureus* (MRSA) and for vancomycin-resistant Enterococci (VRE). A large proportion of samples yielded carbapenemase- or ESBL-producing *Enterobacteriaceae* (16% and 55%, respectively), while only two MRSA and two VRE were detected. Mating-out assays followed by PCR and whole-plasmid sequencing allowed to identify carbapenemase and ESBL encoding genes. Among 24 carbapenemase-producing isolates, there were mainly *Klebsiella pneumoniae* (50%) and *Escherichia coli* (33%). OXA-181 was the most common carbapenemase identified (54%), followed by OXA-48 (25%) and KPC-2 (17%). Ten different ESBLs were found among 62 ESBL-producing isolates, mainly being CTX-M-type enzymes (87%). Co-occurrence in single samples of multiple ESBL- and carbapenemase producers belonging to different bacterial species was observed in some cases. Seagulls constitute an important source for spreading multidrug-resistant bacteria in the environment and their gut microbiota a formidable microenvironment for transfer of resistance genes within bacterial species.

## 1. Introduction 

*Enterobacteriaceae* producing extended-spectrum β-lactamases (ESBL) and/or carbapenemases are not only a major concern in the healthcare setting (mostly *Klebsiella pneumoniae*), but also frequently recovered, mainly as colonizers, in the community, among livestock, and in the environment (mostly *Escherichia coli*). Wild birds are known to be carriers of antibiotic-resistant bacteria, and studies focusing on their microbiota contribute to evaluate the nature and extent of spread of corresponding genes in the environment. Of note, those birds can therefore constitute significant reservoirs of resistant pathogens and significant spreaders through migration [1]. In recent years, gulls have been reported as carriers of ESBL-producing *E. coli* in Europe, South America, North America, and Asia [2,3,4,5,6]. More recently, a very limited number of studies documented fecal carriage of carbapenemase-producers in gulls in Spain, France, Australia, and Alaska, being exclusively *E. coli* [7,8,9,10]. 

Multidrug resistance is a major concern in Portuguese hospitals: (a) the prevalence of ESBL producers in invasive *K. pneumoniae* was estimated as 50% in 2018, considerably overcoming the European rate (31.7%); (b) in recent years, there has been a notorious increasing trend in carbapenem resistance among *K. pneumoniae* (from 1.8% in 2014 to 11.7% in 2018); (c) the prevalence of methicillin-resistant *Staphylococcus aureus* (MRSA) is still one of the highest in Europe (38.1%); (d) while a decreasing trend has been concomitantly observed with vancomycin-resistant Enterococci (VRE) and, in particular, *Enterococcus faecium* (from 20.3% in 2015 to 4.4% in 2018) [11]. 

On the other hand, some studies performed in Portugal looked at the prevalence of ESBL producers in gull feces, reporting values between 12% and 19% [2,12,13] within gull populations and a predominance of ESBL CTX-M-14 in the Algarve (South), CTX-M-15 and CTX-M-32 in Oporto (North), and TEM-52 in the natural reserve of Berlengas Island. None of these studies were conducted in the Lisbon area, and none of those studies investigated the occurrence of carbapenemase producers. 

The first clinical carbapenemase-producing *K. pneumoniae* (KPC-3 producer) isolated in Portugal had been recovered from a patient hospitalized in Lisbon in 2009 [14]. Contemporary carbapenemase-producing *Enterobacteriaceae* in the country produced mainly KPC-3, KPC-2, OXA-48, and OXA-181 enzymes [15,16,17]. GES-5 was mainly found sporadically with the exception of one hospital in Lisbon, where it represented 17% of the carbapenemase-producing *K. pneumoniae* isolates [16]. Although NDM producers have been recently detected in a single patient in Lisbon [18], they are still infrequent in the country.

The aim of the present study was to investigate the intestinal carriage of multidrug-resistant human pathogens in gulls present along the Lisbon coastline, Portugal, including *Enterobacteriaceae* producing ESBL and/or carbapenemases, MRSA, and VRE.

## 2. Materials and Methods

### 2.1. Sample Collection and Bacterial Isolates 

In November 2019, a total of 88 fresh gull fecal samples were collected at three areas in the Lisbon coastline, Portugal (Caxias, *n* = 23; Paço d’Arcos, *n* = 41; and Carcavelos, *n* = 24), located ~5 km apart of each other. To minimize repeated sampling, the recovery of specimens was performed on the same day for each sampling area. In order to prevent cross-contamination, only wet, fresh, and separate feces were collected into sterile plastic tubes. Samples were incubated overnight at 37 °C in Tryptic Soy Broth (Becton, Dickinson & Co, Franklin Lakes, NJ, USA) for non-selective pre-enrichment. 

The next day, a volume of 25 μL of each broth was inoculated onto four different selective media: (i) CHROMagar ESBL (Frilabo, Maia, Portugal) to select ESBL-producing Gram-negatives; (ii) ChromID Carba Smart selective medium (bioMérieux, La Balme-les-Grottes, France) to select carbapenem-resistant Gram-negatives; (iii) Chromagar MRSA (ChromAgar, Paris, France) to select MRSA, and (iv) Chromagar VRE (ChromAgar) to select VRE. A single colony per morphotype and per selective plate was picked up for further analysis.

Enterobacterial isolates were identified at the species level using the API20E system (bioMérieux). *S. aureus* species identification was confirmed by PCR amplification of the *spa* gene [19]. 

### 2.2. Antimicrobial Susceptibility Testing 

Antimicrobial susceptibility testing was performed on all *Enterobacteriaceae* isolates by using the disc diffusion method on Mueller–Hinton (MH) agar plates (Bio-Rad, Cressier, Switzerland) for ticarcillin, amoxicillin/clavulanic acid, ceftazidime, cefoxitin, cefotaxime, temocillin, imipenem, ertapenem, meropenem, gentamicin, amikacin, tobramycin, ciprofloxacin, trimethoprim-sulfamethoxazole (SXT), tetracycline, and fosfomycin, following EUCAST 2020 recommendations and breakpoint tables.

### 2.3. Identification of Resistance Determinants

Identification of carbapenemase genes [20] and ESBL genes [21] was performed by PCR amplification using specific primers as described previously, followed by sequencing of the amplicons. We used standard PCR conditions to amplify the β-lactamase gene *bla*_CMY_, encoding plasmid-mediated cephalosporinases [22]. Fosfomycin resistance due to a transferable glutathione-*S*-transferase FosA-like enzyme was searched by using the phosphonoformate test [23] after which plasmid-borne fosfomycin resistance determinants were identified by PCR followed by sequencing [24,25]. 

Detection of the *mecA* [26], *vanA*, and *vanB* [27] genes was performed by PCR on isolates that grew on selective media for MRSA and VRE, respectively.

### 2.4. Molecular Typing

The clonal relationship of all carbapenemase producers was evaluated by pulsed-field gel electrophoresis (PFGE), as described previously [28]. Multilocus sequence typing (MLST) was performed for all *K. pneumoniae* and *E. coli* isolates, and sequence types (STs) were assigned using the MLST databases for *K. pneumoniae* (https://bigsdb.pasteur.fr/klebsiella/) and *E. coli* (http://enterobase.warwick.ac.uk/species/ecoli/allele_st_search). The genetic relatedness of the MRSA isolates was investigated by *spa* typing [19]. 

### 2.5. Plasmid Analysis and Mating-Out Assays

Transferability of the carbapenem resistance determinants was demonstrated by mating-out assays on representative isolates. Briefly, the respective donors and the azide-resistant *E. coli* recipient strain J53 were separately inoculated overnight in LB broth. Samples were then mixed at a ratio of 10:1 (recipient:donor) for 3 h at 37 °C and plated onto LB agar plates supplemented with azide (100 µg/mL) and ceftazidime (30 µg/mL). Antimicrobial susceptibility testing was performed on the *E. coli* transconjugants in order to identify putative co-resistances. Kieser extraction [29] followed by gel electrophoresis analysis were performed for the resulting *E. coli* transconjugant strains in order to estimate the size of the transferred plasmid. *E. coli* strain ATCC50192 carrying four plasmids with known sizes (7 kb, 48 kb, 66 kb, and 154 kb) was used as molecular marker.

PCR-based replicon typing (PBRT) was performed on DNA recovered from *E. coli* transconjugants to identify specific replicase genes eventually differentiating plasmid incompatibility groups [30]. 

### 2.6. Plasmid Sequencing and Bioinformatic Analysis

Sequencing of plasmid DNAs from transconjugants was performed by using an Illumina technology. Templates used corresponded to plasmid extracts obtained by using the Qiagen Large Plasmid Construct kit (Qiagen, Hilden, Germany) for three isolates (*K. pneumoniae* 39E A and 25CAR A and *E. coli* 81OXA R). Genomic libraries were assessed using a Nextera XT library preparation kit (Illumina Inc., San Diego, CA, USA), and sequencing was performed using an Illumina MiniSeq system with 150-bp paired-end reads and a coverage of 50 times. The generated FastQ data were compiled and analyzed using the CLC Genomic Workbench (version 7.5.1; CLC Bio, Aarhus, Denmark). Reads were de novo assembled with automatic bubble and word size, and contigs with a minimum contig length of 800 nucleotides were generated using the mapping mode map reads back to contigs. The resulting contigs were uploaded into the Center for Genomic Epidemiology server (http://www.genomicepidemiology.org/). The plasmid replicon type was determined using the PlasmidFinder program (version 1.3) and antimicrobial resistance was analyzed by CARD (https://card.mcmaster.ca). 

The plasmid of isolate 25CAR A was constructed with the help of SnapGene (version 5.1), of blastn (https://blast.ncbi.nlm.nih.gov/Blast.cgi), and of Kablammo software for the visualization of BLAST results (http://kablammo.wasmuthlab.org). The constructed pKP25CA-KPC plasmid was annotated by RAST server (https://rast.nmpdr.org).

## 3. Results 

From the 88 gull fecal samples inoculated on the different selective media, a total of 86 enterobacterial isolates showing a phenotypic antibiotic resistance profile compatible with the production of ESBLs or carbapenemases were recovered.

### 3.1. Isolation of Carbapenemase-Producing Enterobacteriaceae 

Among the 88 gull fecal samples, 14 (16%) yielded carbapenemase-producing *Enterobacteriaceae*. Notably, this corresponds to almost half (48%) of the samples recovered at Caxias, while no carbapenem-producing isolate was found at Carcavelos (Table 1). Eight out of the 14 (57%) carbapenemase-positive fecal samples actually grew more than one type of carbapenemase-producing isolate, leading to a total of 24 carbapenemase-producing isolates (Table 2). Those isolates were identified as *K. pneumoniae* (12 isolates; 50%), *E. coli* (8 isolates; 33%), *Citrobacter freundii* (2 isolates), *Enterobacter cloacae* (1 isolate), and *Klebsiella oxytoca* (1 isolate). 

The most commonly identified carbapenemase was OXA-181 (*n* = 13; 54%), followed by OXA-48 (*n* = 6; 25%), KPC-2 (*n* = 4; 17%), KPC-3 (*n* = 1), GES-5 (*n* = 1), and GES-6 (*n* = 1) (Table 2). Two *bla*_OXA-48_-positive isolates co-harbored another carbapenemase gene (*bla*_KPC-2_ or *bla*_GES-5_). Of note, all isolates producing OXA-181 were from Caxias beach, while the two GES- and most (4/5) of the KPC-producing isolates were recovered at Paço d’Arcos (Figure S1). Seven carbapenemase-producing isolates, mainly OXA-181-producing *E. coli*, co-produced an ESBL (CTX-M-15). 

Mating-out assays followed by PBRT revealed that the *bla*_OXA-181_ and *bla*_OXA-48_ genes were always located on IncX3 and IncL plasmids, respectively, while the *bla*_KPC-2_ gene was located on a plasmid belonging to the IncF incompatibility group. The *bla*_GES-5_ and *bla*_GES-6_ genes were both located on ColE1-like plasmids. 

Complete sequencing of plasmid pKP25CA-KPC from strain *K. pneumoniae* 25CAR A was 136,244-kb in-size and belonged to the IncFII incompatibility group. This plasmid harbored a series of resistance genes, namely *bla*_KPC-3_, *bla*_OXA-9_, *bla*_TEM-1_, *sul2*, *aac(6′)-Ib/aac(6′)-II*, *ant(3′’)-Ia*, *aph(3′)*, and *aph(6)-Ic/aph(6)-Id*. Detailed in-silico analysis of pKP25CA-KPC revealed a high identity (respectively 99.74%, 96.62%, and 99.93%) with parts of three different plasmids, namely pKLP268-2 (GenBank no. CP043048), pKpQIL (GenBank no. GU595196), and pKOX3-P2-OXA (GenBank no. KY913898). pKP25CA-KPC shared a significant identity with the backbone of pKpQIL (59%), including the *repA* replicase gene, the replicon partitioning and stabilization genes, SOS system inhibition genes (*psiA*, *psiB*), and individual conjugation-related genes as well as other proteins (Figure S2). Although plasmids pKLP268-2 and pKpQIL harbored transposon Tn*4401* containing the *bla*_KPC-3_ gene, the Tn*4401a* isoform was identified in plasmid pKP25CA-KPC. That latter carried a conjugation transfer region and the replicase *repA* of pKpQIL and pKOX3-P2-OXA, the trimethoprim resistance gene *dfrA14*, the *sul2* gene, and four aminoglycoside resistance genes as for pKLP268-2. pKP25CA-KPC also harbored the *czcD* gene coding for a cobalt–zinc–cadmium efflux pump identical to that of pKOX3-P2-OXA. 

PFGE and MLST showed significant genetic diversity (Table 2). The 12 *K. pneumoniae* isolates were distributed into nine PFGE types and seven STs, while the eight *E. coli* isolates belonged to seven PFGE types and five STs. Among *E. coli* isolates, there was a major clone, namely ST940 (4 isolates; 50%), while three STs (ST17, ST321, and ST4845) were identified among *K. pneumoniae* isolates. 

Notably, several carbapenemase-positive fecal samples yielded different bacterial species or different carbapenemases (Table 2). Seven out of the 14 (57%) samples contained more than one species (*K. pneumoniae*/*E. coli* (*n* = 59); *E. cloacae*/*C. freundii* (*n* = 1); *K. oxytoca*/*E. coli* (*n* = 1)). One fecal sample contained three carbapenemase producers and four different carbapenemases (GES-5, GES-6, OXA-48, KPC-2). Furthermore, two samples contained two distinct carbapenemase-positive isolates (*bla*_OXA-48_/*bla*_KPC-2_ and *bla*_OXA-48_/*bla*_GES-5_).

Antimicrobial susceptibility testing showed the following non-susceptibility rates: amoxicillin/clavulanic acid (*n* = 24; 100%), temocillin (*n* = 20; 83%), ertapenem (*n* = 17; 71%), ceftazidime (*n* = 13; 54%), ciprofloxacin (*n* = 13; 54%), tobramycin (*n* = 11; 46%), fosfomycin (*n* = 9; 38%), SXT (*n* = 9; 38%), cefotaxime (*n* = 9; 38%), gentamicin (*n* = 9; 38%), tetracycline (*n* = 7; 29%), cefoxitin (*n* = 6; 25%), imipenem and meropenem (*n* = 2), and amikacin (*n* = 1). The two isolates being non-susceptible to imipenem and meropenem were a single *K. oxytoca* (KPC-2 producer) and a single *C. freundii* (producing both OXA-48 and KPC-2). Overall, a significant number of carbapenemase-producing but ertapenem-susceptible isolates of *E. coli* was identified. 

### 3.2. Isolation of ESBL-Producing Enterobacteriaceae 

Among the 88 fecal samples, 48 (55%) were positive for ESBL-producing *Enterobacteriaceae* (carbapenemase negative) (Table 1). The highest prevalence of ESBL-producing isolates was found in samples from Caxias (83%), followed by Paço d’Arcos (54%), and Carcavelos (29%), mirroring the relative abundance of carbapenemase-producing isolates in those different geographical sites.

Twelve samples contained more than one ESBL-producing isolate, leading to 62 ESBL-producing isolates in total. Those isolates were identified as *E. coli* (39 isolates; 63%), *K. pneumoniae* (21 isolates; 34%), and *E. cloacae* (2 isolates).

The genotypic characterization identified 10 different ESBLs (Table 3), being mainly CTX-M-type enzymes (54/62; 87%). CTX-M-15, representing 53% of the ESBLs (*n* = 33), was identified in the three sampling areas and was particularly predominant in Caxias, while CTX-M-1 was mainly found in Paço d’Arcos (Figure S3). Two *E. coli* isolates producing CTX-M-15 or SHV-12, co-harbored CMY-type AmpC β-lactamase encoding genes.

More than half of the isolates were resistant to cefotaxime (*n* = 48; 77%), ceftazidime (*n* = 43; 69%), tetracycline (*n* = 38; 61%), SXT (*n* = 37; 60%), and ciprofloxacin (*n* = 36; 58%). In addition, resistance was observed for tobramycin (*n* = 20; 32%), amoxicillin/clavulanic acid (*n* = 16; 26%), gentamicin (*n* = 14; 23%), fosfomycin (*n* = 13; 21%), cefoxitin (*n* = 6; 10%), and temocillin (*n* = 1). None of the isolates showed resistance to amikacin. A single *E. coli* isolate showing high level resistance to fosfomycin carried the transferable *fosA3* gene. 

### 3.3. Isolation of MRSA and VRE 

Two samples carrying an ESBL-producing *E. coli* isolate also yielded MRSA isolates. These two MRSA belonged to *spa* types t008 and t121, associated to clonal complex CC8. Additionally, two samples carried concomitantly one ESBL-producing *E. coli* and a *vanA*-positive VRE.

## 4. Discussion

Our study revealed frequent occurrence of carbapenemase- and ESBL-producing *Enterobacteriaceae* (16% and 55%, respectively) colonizing gulls from the Lisbon coastline, Portugal. ESBL-producing *Enterobacteriaceae* among gulls were previously reported in USA [6], Canada [4], Chile [5], and in different countries in Europe [8,31,32,33,34], including Portugal in 2008 [12,13]. The high heterogeneity observed here in terms of bacterial species, ST, and resistance determinants showed that the high resistance rates observed were basically not simply consequences of shared microbiota between bird individuals, that could have biased our observation.

A few studies reported carbapenemase-producing *Enterobacteriaceae* from gulls, with a higher prevalence in Australia (40%) and France (19%) [7,9] and lower rates in Spain (1.5%) and Alaska (<1%) [8,35]. Our study provided original observations, among which the high number of ESBL- and carbapenemase-producing *K. pneumoniae* is notorious. Indeed, this contrasts with previous studies that identified those resistance traits exclusively in *E. coli*, which was somehow expected owing to the infrequent occurrence of *K. pneumoniae* in animals and in the environment compared to *E. coli*. Our findings are also noteworthy in terms of diversity of carbapenemase enzymes (OXA-181, OXA-48, KPC-2, KPC-3, GES-5, and GES-6), although previous studies so far reported carbapenemases IMP-4 and VIM-1 (respectively in Australia and France) in gulls. 

Remarkably, some samples were positive for multiple ESBL- and/or carbapenemase-encoding genes (one sample being positive for two carbapenemase producers each producing two different carbapenemases, for a total of four distinct carbapenemases), identified from different bacterial species. Moreover, a high prevalence of tetracycline and ciprofloxacin resistances was observed, while susceptibility rates to aminoglycosides remained overall quite low. Of note, only a single *K. pneumoniae* isolate was found resistant to fosfomycin, showing that this critical antibiotic for treating urinary tract infection showed an excellent activity overall. Regarding the different plasmid types identified, those at the origin of the acquisition of *bla*_OXA-48_ and *bla*_OXA-181_, being respectively IncL and IncX3, actually corresponded to the known epidemic ones worldwide. Hence, the gull’s gut microbiota constitutes a potential micro-environment where transfer of resistance genes between bacterial species may be highly facilitated. 

As observed in previous studies, the isolates colonizing gulls showed significant similarities with human clinical isolates. Indeed, all types of ESBLs and carbapenemases identified in this study were also found among human samples in Portugal. The most common acquired carbapenemases identified from clinical samples in the country are KPC (KPC-2 and KPC-3) and OXA-48-type enzymes (OXA-48 and OXA-181) [15,16,17]. GES-5 and GES-6 have been found sporadically, with the exception of one hospital in Lisbon where *K. pneumoniae* isolates co-harboring *bla*_KPC-3_ and *bla*_GES-5_ represented 17% of the isolates [16]. Although NDM producers have been recently detected in a single patient in Lisbon [18], no *bla*_NDM-1_ was found in gulls in our study, suggesting its still low diffusion rate. While ST940 was the predominant *E. coli* background identified from gulls in our study, this ST had not been identified in Portugal before and, to the best of our knowledge, has been exclusively identified among NDM-5-producing *E. coli* isolates in the US [36]. By contrast, the *K. pneumoniae* ST17 background that has frequently been identified here is a clonal type frequently found among clinical isolates recovered from Portuguese patients [24]. This ST was also identified in other countries [37], and not only among hospitalized patients but also in healthy individuals and companion animals [38]. 

The principal ESBL identified in this study was CTX-M-15, which corresponds to the predominant ESBL found among clinical *Enterobacteriaceae* in Portugal [24,39,40] and worldwide [41]. This ESBL was actually massively detected among healthy pigs in Portugal [42], either in *E. coli* or *K. pneumoniae*. Likewise, ESBLs CTX-M-1 and SHV-12, also well represented in our collection, were frequently identified among Portuguese human isolates [43]. Consequently, we may speculate that the ESBL and carbapenemase producers identified among gull feces might correspond to strains circulating among humans.

Notably, only two MRSA and two VRE isolates were detected here. This low rate might be explained by the fact the prevalence of invasive vancomycin-resistant *E. faecium* in Portugal has considerably dropped during the last years (20.3% in 2015 to 4.4% in 2018). On the other hand, and despite the prevalence of invasive MRSA in Portugal being still one of the highest in Europe (38.1%) [11], few MRSA isolates were identified here. However, it is known that the intestinal track (at least that of humans) does not represent a major reservoir for MRSA, that could explain this low rate here. Nevertheless, the two MRSA isolates identified here belonged to the major clone circulating in hospitals all over the country and for a long time [44]. 

Overall, the clinically relevant bacteria identified in our study seem to qualitatively mirror the human panorama, either in terms of diversity of β-lactamases circulating in this specific geographical area, but also in terms of clonal backgrounds. This might be considered as unsurprising considering that although gulls are marine feeders, they also use food sources provided by humans, especially garbage, and drink environmental water (including from wastewater treatment plants) that may be contaminated by human gut colonizers. 

A very recent work that assessed the occurrence of carbapenem-resistant *Enterobacteriaceae* in a river in Portugal in samples collected near a wastewater treatment plant and livestock farms, detected *bla*_KPC-3_ in *K. pneumoniae* (*n* = 9), *bla*_NDM-1_ in *Enterobacter* (*n* = 3), and *bla*_GES-5_ in *Citrobacter* (*n* = 1), evidencing effluents as important reservoirs of multidrug resistant human pathogens, including *Enterobacteriaceae* [45]. In our study, gulls from Caxias were heavily colonized compared with gulls from other areas of the coastline. Of note, there is an urban effluent arriving at Caxias beach, with a history of poor quality due essentially to discharges of rainwater of urban origin and clandestine domestic wastewater discharges [46]. This might explain the higher rates of ESBL- and carbapenemases-producing isolates among gulls in this area.

Since our study relied on a limited number of sampling sites and a limited number of feces specimen, it would be important to extend such survey by enlarging the sample size in future studies which would subsequently allow to better evaluate the extent of such threatening observation.

## Figures and Tables

**Table 1:** Samples recovered, and samples in which carbapenemase or ESBL producers were recovered.

Sampling Area	Samples Recovered	Samples Positive for Carbapenemase Producers		Samples Positive for ESBL Producers	
		No.	%	No.	%
Carcavelos	24	0	0%	7	29%
Paço d’Arcos	41	3	7%	22	54%
Caxias	23	11	48%	19	83%
Total	88	14	16%	48	55%

**Table 2:** Characteristics of the 24 carbapenemase-producing *Enterobacteriaceae* isolates.

Fecal Sample	Sampling Area	Isolate	Species	PFGE	MLST	Resistance Genes ^a^	Plasmid Type ^b^	TIC	CAZ	AMC	CTX	ETP	IMP	MEP	TEM	FOX	CIP	SXT	FOS	TET	AMK	GEN	TOB
81	Caxias	81OXA R	*E. coli*	A	ST219	*bla* _OXA-48_	IncL	R	S	R	S	R	S	S	R	S	S	S	S	S	S	S	S
82	Caxias	82OXA R	*E. coli*	B	ST80	*bla* _OXA-48_	IncL	R	S	R	S	S	S	S	R	S	S	S	S	S	S	S	S
85	Caxias	85OXA R	*E. coli*	C	ST940	*bla* _OXA-181_	IncX3	R	S	R	S	S	S	S	R	S	S	S	S	R	S	S	S
79	Caxias	79CAR R	*E. coli*	D	ST940	*bla* _OXA-181_	IncX3	R	S	R	S	S	S	S	R	S	S	S	S	R	S	S	S
80	Caxias	80OXA R	*E. coli*	D	ST940	*bla* _OXA-181_	IncX3	R	S	R	S	S	S	S	R	S	S	S	S	R	S	S	S
79	Caxias	79OXA R	*E. coli*	E	ST940	*bla*_OXA-181_, *bla*_CTX-M-15_	IncX3	R	R	R	R	R	S	S	R	R	R	S	S	R	S	S	S
88	Caxias	88OXA R	*E. coli*	F	SLV ST4440	*bla* _OXA-181_	IncX3	R	S	R	S	S	S	S	R	S	S	S	S	S	S	S	S
72	Caxias	72OXA R	*E. coli*	G	ST155	*bla* _OXA-181_	IncX3	R	S	R	S	S	S	S	R	S	S	S	S	S	S	S	S
77	Caxias	77OXA A	*K. pneumoniae*	J	ST321	*bla* _OXA-48_	IncL	R	S	R	S	R	S	S	R	S	I	S	S	S	S	S	S
79	Caxias	79OXA A	*K. pneumoniae*	J	ST4845	*bla* _OXA-181_	IncX3	R	S	R	S	R	S	S	R	S	S	S	R	S	S	S	S
85	Caxias	85OXA A	*K. pneumoniae*	J	ST321	*bla* _OXA-181_	IncX3	R	S	R	S	R	S	S	R	S	I	S	S	S	S	S	S
86	Caxias	86OXA A	*K. pneumoniae*	J	ST321	*bla* _OXA-181_	IncX3	R	S	R	S	R	S	S	R	S	R	S	R	S	S	S	S
83	Caxias	83OXA A	*K. pneumoniae*	I	ST17	*bla*_OXA-181_, *bla*_CTX-M-15_	IncX3	R	R	R	R	R	S	S	R	S	R	R	R	S	S	R	R
72	Caxias	72OXA A	*K. pneumoniae*	K	ST17	*bla*_OXA-181_, *bla*_CTX-M-15_	IncX3	R	R	R	R	R	S	S	R	S	R	R	R	R	S	S	R
70	Caxias	70OXA A	*K. pneumoniae*	O	ST17	*bla*_OXA-181_, *bla*_CTX-M-15_	IncX3	R	R	R	R	R	S	S	R	R	R	R	R	S	S	R	R
80	Caxias	80OXA A	*K. pneumoniae*	L	ST4845	*bla*_OXA-48_, *bla*_CTX-M-15_	IncL	R	R	R	R	R	S	S	R	S	R	R	R	R	S	R	R
88	Caxias	88OXA A	*K. pneumoniae*	M	New 1	*bla*_OXA-181_, *bla*_CTX-M-15_	IncX3	R	R	R	R	R	S	S	R	S	R	R	S	S	S	S	R
25	Paço d’Arcos	25CAR A	*K. pneumoniae*	P	New 2	*bla* _KPC-3_	IncFII	R	R	R	S	R	S	S	S	S	I	R	S	S	S	R	R
44	Paço d’Arcos	44CAR A	*K. pneumoniae*	N	ST13	*bla* _KPC-2_	IncF	R	R	R	R	R	S	S	S	S	S	R	R	S	R	R	R
44	Paço d’Arcos	44E A	*K. pneumoniae*	Q	ST1490	*bla*_KPC-2_, *bla*_CTX-M-15_	IncF	R	R	R	R	R	S	S	S	S	R	R	S	R	S	R	R
45	Paço d’Arcos	45OXA A	*C. freundii*	S	-	*bla* _OXA-48_, *bla*_GES-5_	IncL, ColE1	R	R	R	S	R	S	S	R	R	S	S	S	S	S	R	R
45	Paço d’Arcos	45OXA R	*C. freundii*	T	-	*bla* _OXA-48_, *bla*_KPC-2_	IncL, IncF	R	R	R	I	R	I	I	R	R	I	S	R	S	S	R	R
45	Paço d’Arcos	45CAR A	*E. cloacae*	R	-	*bla* _GES-6_	ColE1	R	R	R	S	S	S	S	S	R	S	S	R	S	S	S	S
81	Caxias	81CAR A	*K. oxytoca*	H	-	*bla* _KPC-2_	IncF	R	R	R	S	R	I	R	R	R	R	R	S	S	S	R	R

ST—Sequence type determined by multilocus sequence typing; TIC—Ticarcillin; CAZ—Ceftazidime; AMC—Amoxicillin/clavulanic acid; CTX—Cefotaxime; ETP—Ertapenem; IMP—Imipenem; MEM—Meropenem; TEM—Temocillin; FOX—Cefoxitin; CIP—Ciprofloxacin; SXT—Trimethoprim-sulfamethoxazole; TET—Tetracycline; AKN—Amikacin; GMI—Gentamicin; TOB—Tobramycin. R—Resistant; I—Intermediate resistant; S—Susceptible. ^a^ Carbapenemase genes are underlined. ^b^ Type of the plasmid carrying the carbapenemase gene.

**Table 3:** Characteristics of the 62 ESBL-producing *Enterobacteriaceae* isolates.

Species	No. of Isolates	ESBL	*bla*_CMY_	TIC	CAZ	AMC	CTX	TEM	FOX	CIP	SXT	FOS	TET	AMK	GEN	TOB
*E. coli* (*n* = 39)	12	CTX-M-15		R	R(10)	S	R	S	S	S(6)	S(6)	S(9)	R(7)	S	S(9)	S(9)
			1	R	R	R	R	S	R	S	R	S	S	S	S	S
	8	CTX-M-1		R	S(6)	S(7)	R	S	S(7)	S(5)	S(5)	S	R(5)	S	S	S
	7	SHV-12		R	R	S	S	S	S	S	S(4)	S	R(4)	S	S	S
			1	R	R	R	S	S	R	R	S	S	S	S	S	S
	4	CTX-M-65		R	S(3)	S(3)	R	S(3)	S(3)	R	S(2)	S(3)	S(2)	S	S	R(3)
	3	CTX-M-32		R	R (2)	S	R	S	S	R(2)	S(2)	S	R(2)	S	S	S
	2	CTX-M-55		R	R	S	R	S	S	R	R	S	R	S	S	S
	1	CTX-M-8		R	S	S	S *	S	S	R	S	S	R	S	R	R
	1	CTX-M-14		R	S	S	S *	S	S	R	S	S	R	S	S	S
	1	CTX-M-27		R	S	S	S *	S	S	R	S	S	S	S	S	S
*K. pneumoniae* (*n* = 20)	19	CTX-M-15		R	R (16)	S (10)	R	S	S	R(12)	R(17)	S(10)	R(11)	S	R(9)	R(10)
	1	CTX-M-55		R	R	R	R	S	S	R	R	S	S	S	S	R
	1	SHV-2		R	S	S	S	S	S	R	R	R	R	S	S	R
*E. cloacae* (*n* = 2)	2	CTX-M-15		R	R	R	R	S	R	R	R	S	S(1)	S	R	R

* Reduced susceptibility.

## Supplementary Materials

The following are available online at https://www.mdpi.com/2076-2607/8/10/1487/s1, Figure S1: Number of carbapenemase producers (with corresponding carbapenemase types in color) detected on each respective sampling site. Figure S2: Genetic map comparing structural features of plasmid pKP25CA-KPC with sequences of reference plasmids pKLP268-2 (GenBank accession no. CP043048), pKpQIL (GenBank accession no. GU595196) and pKOX3-P2-OXA (GenBank accession no. KY91389). Figure S3: Number of ESBL producers (with corresponding ESBL types in color) detected on each respective sampling site.
