# Supplementary material for: High Colonization Rate and Heterogeneity of ESBL- and Carbapenemase-Producing Enterobacteriaceae Isolated from Gull Feces in Lisbon, Portugal
Source: Microorganisms. 2020 Sep 28;8(10):1487. doi: 10.3390/microorganisms8101487 (PMC7601013; doi:10.3390/microorganisms8101487)
Supplement: Supplementary file 1 [file microorganisms-08-01487-s001.pdf]

Figure S1

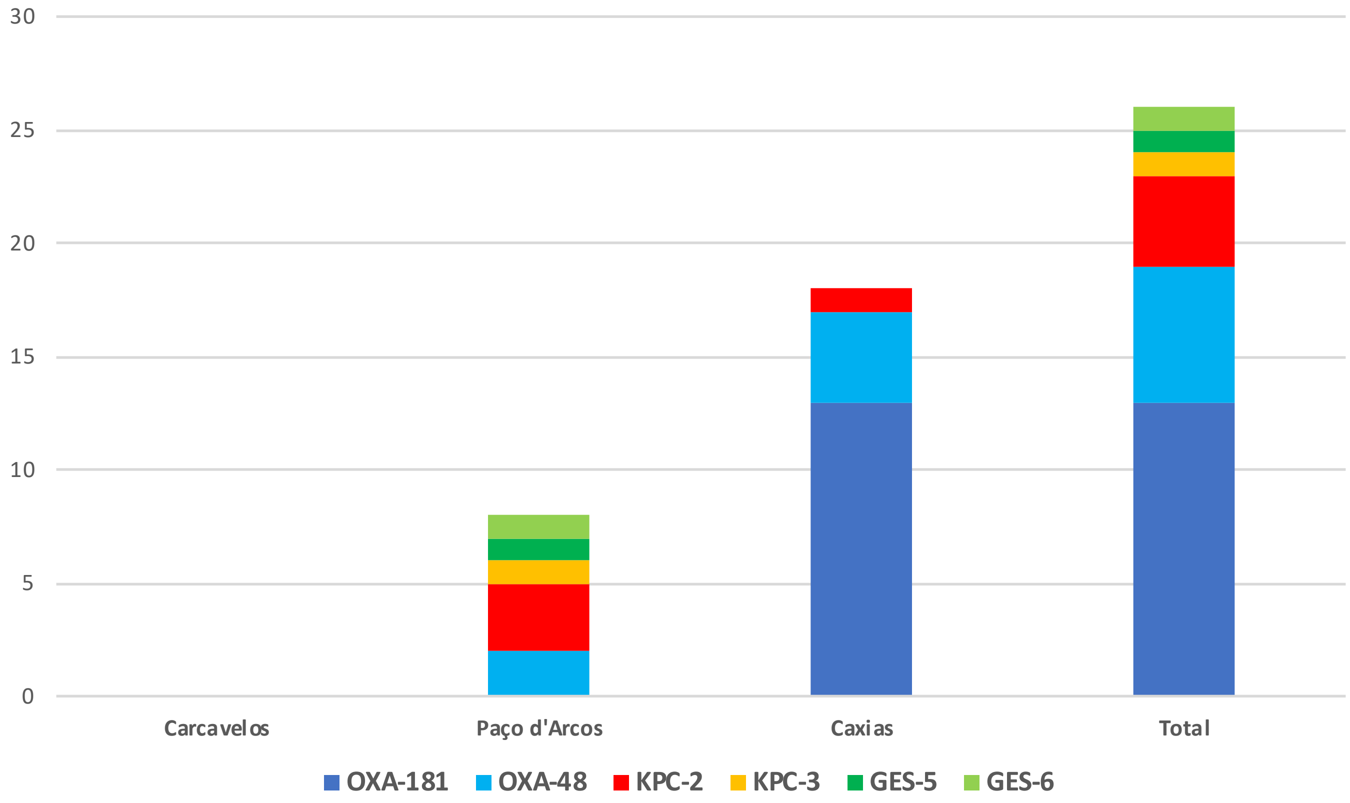
30
25
20
15
10
5
0
Carcavelos
Paço d'Arcos
Caxias
Total
OXA-181
OXA-48
KPC-2
KPC-3
GES-5
GES-6

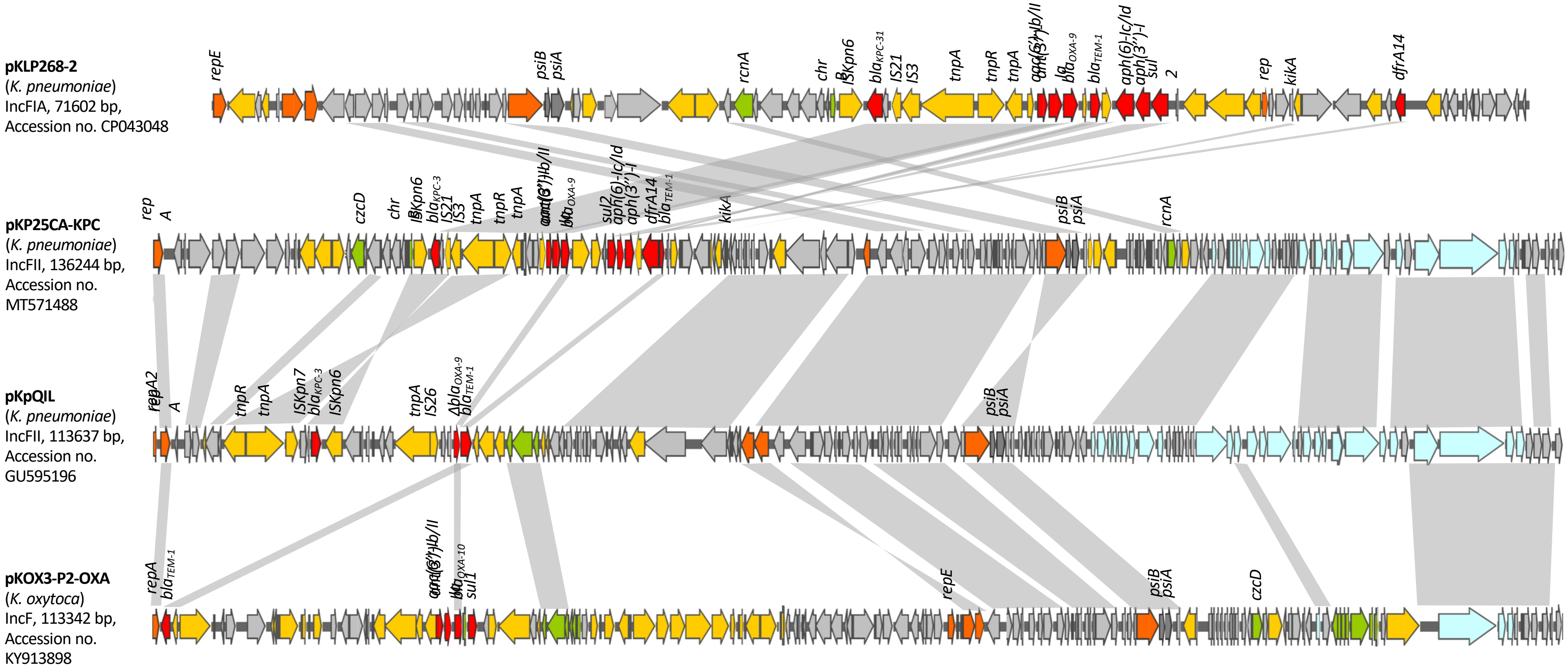

Figure S2. Genetic map comparing structural features of plasmid pKP25CA-KPC with sequences of reference plasmids pKLP268-2 (GenBank accession no. CP043048), pKpQIL (GenBank accession no. GU595196) and pKOX3-P2-OXA (GenBank accession no. KY913898). Gray shading indicates homologies between the corresponding genetic loci on each plasmid. Arrows indicate gene location and direction of the transcription : red, antibiotic resistance encoding genes; green, metal resistance encoding genes; yellow, transposon- and integron-associated genes; orange, replicon, partitioning and stability associated genes; teal, individual conjugation-related genes (associated wit *tra* and *trh*); dark grey, SOS system inhibition genes; grey, other or hypothetical proteins. The figure is not drawn to scale.

Figure S3

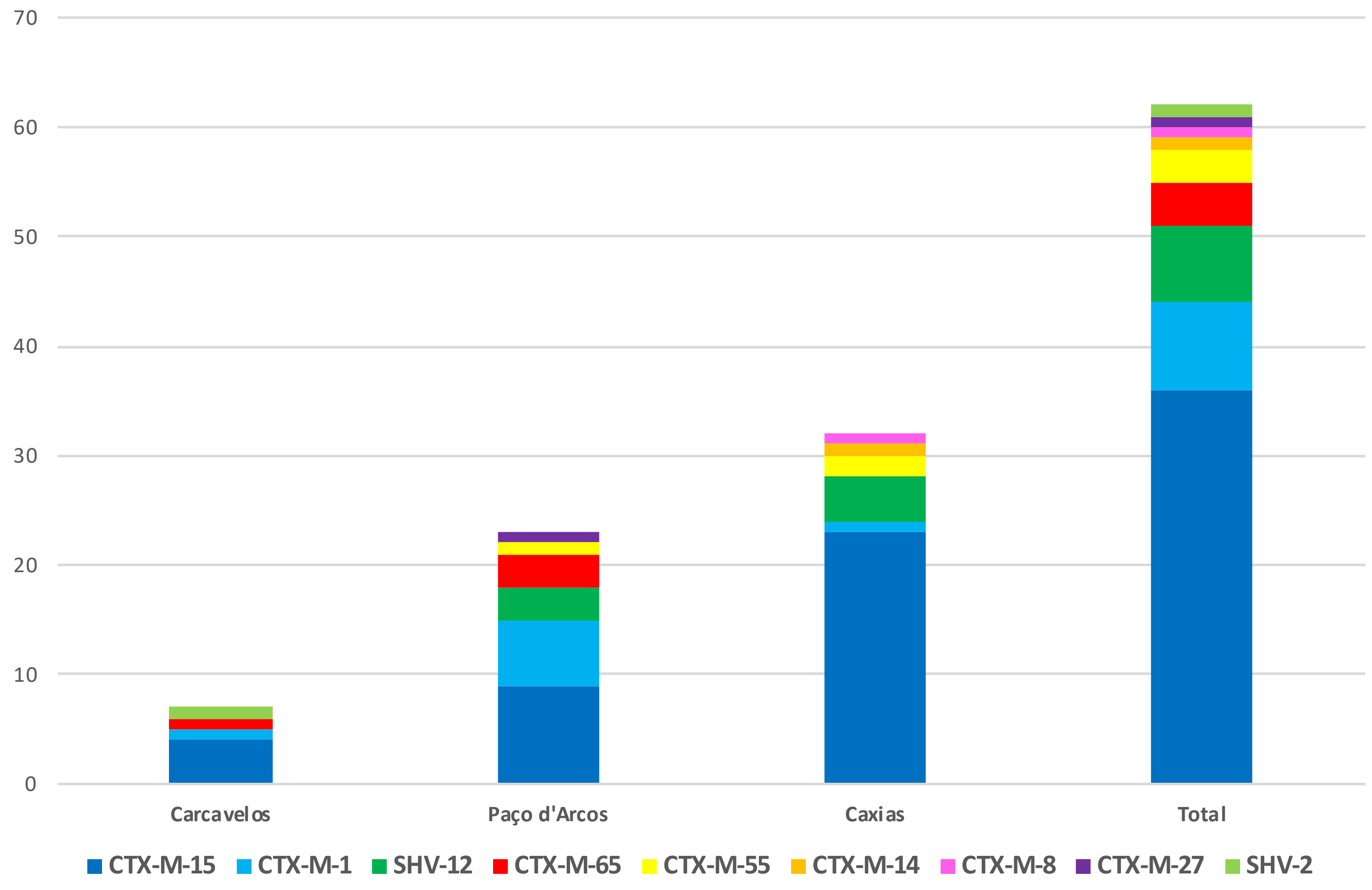
70
60
50
40
30
20
10
0
Carcavelos
Paço d'Arcos
Caxias
Total
CTX-M-15
CTX-M-1
SHV-12
CTX-M-65
CTX-M-55
CTX-M-14
CTX-M-8
CTX-M-27
SHV-2
